# Cortical Circuit Dysfunction as a Potential Driver of Amyotrophic Lateral Sclerosis

**DOI:** 10.3389/fnins.2020.00363

**Published:** 2020-04-29

**Authors:** Aurore Brunet, Geoffrey Stuart-Lopez, Thibaut Burg, Jelena Scekic-Zahirovic, Caroline Rouaux

**Affiliations:** INSERM UMR_S 1118, Mécanismes Centraux et Périphériques de la Neurodégénérescence, Faculté de Médecine, Université de Strasbourg, Strasbourg, France

**Keywords:** amyotrophic lateral sclerosis, cerebral cortex, hyperexcitability, network dysfunction, intrinsic, extrinsic

## Abstract

Amyotrophic lateral sclerosis (ALS) is a devastating neurodegenerative disease that affects selected cortical and spinal neuronal populations, leading to progressive paralysis and death. A growing body of evidences suggests that the disease may originate in the cerebral cortex and propagate in a corticofugal manner. In particular, transcranial magnetic stimulation studies revealed that ALS patients present with early cortical hyperexcitability arising from a combination of increased excitability and decreased inhibition. Here, we discuss the possibility that initial cortical circuit dysfunction might act as the main driver of ALS onset and progression, and review recent functional, imaging and transcriptomic studies conducted on ALS patients, along with electrophysiological, pathological and transcriptomic studies on animal and cellular models of the disease, in order to evaluate the potential cellular and molecular origins of cortical hyperexcitability in ALS.

## Introduction

### Definition and Epidemiology of ALS

Amyotrophic lateral sclerosis (ALS) is the most common adult-onset neurodegenerative disease of the motor neuron. It manifests as an initial focal muscular weakness and progresses into full paralysis of most of the skeletal muscles. ALS leads rapidly to death with a median survival of only 2 to 3 years of diagnosis. ALS was named after the initial histological description made by French neurologist Jean-Martin Charcot in 1869 (Charcot, 1874), who reported two pathological hallmarks in the spinal cord of patients: the degeneration of the corticospinal tract (CST), in the lateral columns (lateral sclerosis), and the disappearance of the spinal motor neurons (amyotrophic), in the ventral horns. This initial histological definition still corresponds to the current clinical definition of the disease. Indeed ALS is diagnosed when signs of corticospinal and corticobulbar neurons (CSN) degeneration (i.e., slowness of motions, hyperreflexia, spasticity), and of bulbar and spinal motor neurons (MN) degeneration (i.e., muscular weakness and atrophy, fibrillations, and fasciculations) are found in combination (Brown and Al-Chalabi, 2017; van Es et al., 2017). Above these precise histological and clinical descriptions, it is now also broadly admitted that ALS is a heterogeneous multisystem disease that also implies frequent extramotor symptoms, and particularly behavioral and cognitive deficits, as well as defective energy metabolism (Dupuis et al., 2011).

ALS is the third most frequent neurodegenerative disease after Alzheimer’s and Parkinson’s diseases, with an incidence of 2.6/100,000 person-years and a prevalence of 7–9/100,000 persons (Hardiman et al., 2017b), which reflects the rapid progression of the disease even if very rare cases exist that evolve over decades. ALS affects mostly people in their late fifties, and is overall 1.5 times more frequent amongst men than women (Brown and Al-Chalabi, 2017; Rosenbohm et al., 2018). The vast majority of patients (90%) are considered sporadic, with no familial history. The remaining 10% instead are familial cases with a usually dominant transmission and a high penetrance (Brown and Al-Chalabi, 2017). More than 120 genetic mutations have been associated to ALS, and amongst those, at least 25 genes related to either familial or sporadic ALS, or both (Brown and Al-Chalabi, 2017). About 70% of familial ALS cases, and as much as 15% of sporadic ALS cases, have now been related to mutations (Chia et al., 2017). The most common causative genes are *C9ORF72* (close to 45% of familial cases), *SOD1* (close to 20% of familial cases), *FUS* and *TARDBP* (about 4% of familial cases each) (Brown and Al-Chalabi, 2017; Chia et al., 2017). These familial cases have greatly contributed to the study of the presymptomatic period, and the identification of the causative genes have and continue to inform the development of cellular and animal models of the disease.

### The Debated Origin of ALS

The duality of the neuronal impairments that characterizes ALS is likely responsible for the greater severity of the disease in comparison with neurodegenerative diseases that target only the CSN (hereditary spastic paraplegia, primary lateral sclerosis) or the MN (Kennedy’s disease, adult-onset spinal and muscular atrophy) (Sorenson, 2012). But this duality also raised the question of the origin of the disease, which remains controversial (Ravits and La Spada, 2009).

For Charcot, ALS was a progressive descending neurodegeneration, initiated in the motor cortex, spreading to the MN and ultimately affecting the neuromuscular junctions (Eisen et al., 1992), a view which was later called the “dying-forward” hypothesis. This view was further supported by typical symptoms revealed by deep clinical examinations, such as gait abnormalities or the split hand syndrome, which are highly suggestive of a cortical origin of the disease (Eisen et al., 1992, 2017). Two alternative scenarios were proposed later: a simultaneous and independent degeneration of CSN and MN – which was quickly abandoned, due to the somatotopic relationship that exists between CSN and MN degenerations – and a retrograde progression of the neurodegeneration, known as the “dying-back hypothesis” (for review, see Eisen and Weber, 2001). With the numerous rodent models that followed the discovery of *SOD1* (Lutz, 2018), and the more recent emergence of induced MN from ALS patients’ iPSC (Guo et al., 2017), the vast majority of preclinical studies have concentrated on deciphering the mechanisms involved in MN degeneration, implicitly favoring the “dying-back” hypothesis.

### Arguments Toward a Cortical Origin

#### Genetic and Clinical Evidences

Recent genetic data have established a clear link between ALS and frontotemporal dementia (FTD), a neurodegenerative disease that affects the frontal and temporal cortices and results in behavioral and cognitive deficits. Mutations on several common genes have been identified in ALS and FTD familial cases (Ling et al., 2013). While the clinical presentations of ALS and FTD are extremely different, about 15% of ALS patients develop over time behavioral and cognitive deficits typical of FTD, and about 15% of FTD patients develop over time motor impairments typical of ALS (Ling et al., 2013). Finally, the link between ALS and FTD is also pathologic: the vast majority of ALS patients, whether of familial or sporadic origin, and as much as 45% of FTD patients present with the so-called TDP-43 pathology, i.e., toxic intra-cytoplasmic inclusions of a phosphorylated and misfolded form of the TDP-43 protein (Neumann et al., 2006; Ling et al., 2013). Altogether, genetic, clinical and pathological data have converged to unravel a strong link between ALS and FTD. Both diseases are thus currently seen as the two extremes of a single clinical continuum, with identified common denominators among which is a shared affected region of the central nervous system, the cerebral cortex, which stresses a potential primary contribution of this structure to both diseases.

#### Histopathological Clues and the Corticofugal Hypothesis

Extensive examination of the TDP-43 pathology in post-mortem brains from ALS patients has shown that the motor cortex is the most affected region of the brain, together with the brain stem and spinal cord (Brettschneider et al., 2013). Importantly, by assessing the extent of the TDP-43 pathology in other brain regions, the authors proposed a “corticofugal hypothesis of ALS,” with an origin of the pathology in the motor cortex, followed by a sequential corticofugal pattern of progression to the downstream targets of the motor cortex via a direct, mono-synaptic transmission, similar to a prion-like mechanism (Braak et al., 2013). Such a corticofugal propagation of neurodegeneration is supported by longitudinal diffusion tensor imaging studies with tractography or connectome analysis of ALS patients (Verstraete et al., 2013; Kassubek et al., 2014). These pathology and imaging data further support a primary impairment of the motor cortex in ALS.

#### Functional Studies and Early Cortical Hyperexcitability

Transcranial magnetic stimulation studies unraveled early hyperexcitability as a marker of cortical dysfunction in sporadic and familial ALS patients (Vucic and Kiernan, 2017). Importantly, longitudinal studies in pre-symptomatic *SOD1* mutation carriers revealed that cortical hyperexcitability develops prior to clinical onset of ALS (Vucic et al., 2008), and characterizes also early sporadic patients (Mills and Nithi, 1997; Zanette et al., 2002; Vucic, 2006; Hardiman et al., 2017a). The fact that cortical hyperexcitability negatively correlates with disease progression and survival (Shibuya et al., 2016) highlights the relevance of early cortical dysfunction to ALS onset and progression. Cortical hyperexcitability has been proposed to translate into glutamatergic excitotoxicity to the downstream targets of CSN, and possibly of the whole corticofugal population (Geevasinga et al., 2016; Eisen et al., 2017; Vucic and Kiernan, 2017), contributing to the degeneration of spinal MN via an anterograde trans-synaptic mechanism (Eisen et al., 1992; Vucic and Kiernan, 2017).

#### First Connection Between Hyperexcitability and Pathology

In a recent article, Weskamp et al. (2019) investigated, in iPSC-derived glutamatergic neurons and in mouse primary cortical neurons, the link between hyperexcitability and TDP-43 pathology (Weskamp et al., 2019). In iPSC-derived glutamatergic neurons, neuronal activity was either induced with the potassium channel blocker tetraethylammonium (TEA) or blocked with the sodium channel blocker tetrodotoxin (TTX). In mouse primary cortical neurons, activity was induced or inhibited by glutamate or GABA, respectively. The authors elegantly demonstrated in the two models that increased neuronal activity was accompanied with increased TDP-43 immunoreactivity (Weskamp et al., 2019). They also demonstrated that hyperexcitability induced alternative splicing of *TARDBP* mRNA, leading to the up-regulation of a short isoform of the transcript. This short TDP-43 (sTDP-43) corresponded to the N-terminal part of the full-length protein, and contained a newly identified nuclear export sequence, leading to its cytoplasmic accumulation (Weskamp et al., 2019). Importantly, similar sTDP43 inclusions were identified within the spinal cord and cortex of sporadic ALS and *C9ORF72* ALS patients (Weskamp et al., 2019). In its whole, this study demonstrated for the first time a pivotal connection between neuronal hyperexcitability and TDP-43 pathology, and further supports the earliness of hyperexcitability in the cascade of pathologic events that characterize ALS.

In this review, we present a collection of studies spanning from clinical neurology to electrophysiology, pathology and transcriptomics, in order to interrogate the potential cellular and molecular origins of cortical hyperexcitability in ALS.

## Evidences for Cortical Circuits Dysfunction in ALS

### Cortical Excitability and Hyperexcitability

The terms “excitability” and “conductivity” were primarily related to the investigation of electrical properties of individual neurons and defined respectively as the capability of a structure to respond to a given electrical stimulus by generating an action potential (excitability) and to propagate it along its membrane (conductivity) (Hodgkin and Huxley, 1952). In this regard, “hyperexcitability” was thus initially considered as a decreased threshold of axonal membrane to respond to a stimulus. This initial definition evolved together with the methods to assess excitability at multiple levels of the nervous system. These span from the study of individual neuronal ion channels *ex-vivo*, to the investigation of entire brain regions with transcranial magnetic stimulation (TMS) in clinical neurology (Bakulin et al., 2016). Consequently, the term “hyperexcitability” adapted to describe more broadly an altered electrical activity of various entities, from ion channels to highly specialized neuronal networks, but still lacks a commonly accepted definition. Indeed, it varies from “the ability to respond to stimuli that normally do not evoke any response” (Bakulin et al., 2016), to “an increased or exaggerated response to a stimulus, which may usually have been expected to evoke a normal response” (Bae et al., 2013), to “the predominance of excitation over inhibition” (Bakulin et al., 2016).

Cortical excitability is critically dependent on healthy, balanced, excitatory and inhibitory components. Under physiological conditions, the ratio of excitation to inhibition is invariant and dynamically controlled by the interaction of neurotransmitters and neuromodulators with cellular receptors, leading to the final activation of neurons (Badawy et al., 2012). In the cerebral cortex, this balance is mainly equilibrated by two neurotransmitters, the excitatory glutamate that acts on N-methyl-d-aspartate (NMDA) and non-NMDA receptors, and the inhibitory gamma-aminobutyric acid (GABA) that binds GABA_*A*_ and GABA_*B*_ receptors (Badawy et al., 2012). Perturbation of the excitatory/inhibitory balance can lead to pathological changes in cortical excitability and to the development of neurological disease (Bozzi et al., 2017; Foss-Feig et al., 2017; Vucic and Kiernan, 2017). First attempts to assess cortical excitability were made some 40 years ago when Merton and Morton successfully stimulated the motor cortex by using transcranial electrical stimulation (TES) and delivered electrical impulses, through the scalp, to the primary motor cortex, activating underlying neurons and inducing the twitch of corresponding muscles (Merton and Morton, 1980). Despite their important achievement, the procedure was extremely painful, and Anthony T. Barker began exploring the use of magnetic fields to replace electrical stimulations. First stable transcranial magnetic stimulation devices were developed in the mid-1980s (Barker and Freeston, 1985). Using TMS, Ly et al. (2019) described cortical excitability as the strength of the response of cortical neurons to a given stimulation reflecting both neuron reactivity and response specificity and therefore constituting a fundamental aspect of human brain function.

### TMS Assessment of Cortical Hyperexcitability in ALS

Commonly observed clinical features in ALS like brisk deep tendon reflexes and spasticity, that occur as a result of CSN failure, and spontaneous muscle twitching (fasciculation) and cramps that occur when MN become affected (Brown and Al-Chalabi, 2017), are considered to result from excessive electrical irritability (reviewed in Kleine et al., 2008). Caramia et al. (1991) conducted one of the first single pulse TMS paradigm (spTMS) studies to investigate excitability changes of motor responses to magnetic brain stimulation in patients with motor impairment (hyperreflexia, spasticity, and weakness) in the contexts of multiple sclerosis, ALS, spino-cerebellar ataxia, primary lateral sclerosis, and brain metastasis. ALS patients presented a particularly low threshold to induce motor evoked potential (MEP) (Caramia et al., 1991). Following spTMS studies confirmed this lower “cortical” or “corticomotor” threshold, early in the disease and an increase of this same threshold as disease progressed (Eisen et al., 1993; Mills and Nithi, 1997; Desiato et al., 2002; Mills, 2003; Prout and Eisen, 2004). Lower cortical threshold suggested that the motor cortex of ALS patients was indeed hyperexcitable, at least at the beginning of the clinical manifestations (Caramia et al., 1991; Zanette et al., 2002).

Conventional paired pulse TMS paradigm (cppTMS), in which two stimuli, conditioning and test, are successively applied, allowed to further dissect components of motor cortex excitability (Kujirai et al., 1992; Nakamura et al., 1997) and provided evidences for the existence of early and late intracortical inhibition (ICI), as well as an intracortical facilitation (ICF) of the motor cortex (Nakamura et al., 1997).

**FIGURE 1:**
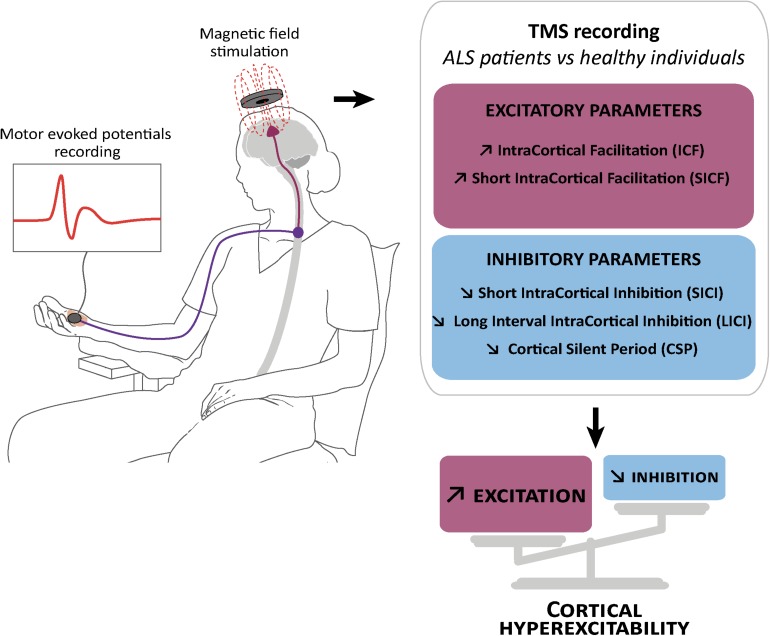
Schematic representation of transcranial magnetic stimulation and cortical hyperexcitability in ALS. Transcranial magnetic stimulation (TMS) consists in the application of a magnetic field above the motor cortex, and the recording of motor evoked potentials in the abductor pollicis brevis muscle of the hand **(left)**. This technique showed reduction or absence of parameters related to cortical inhibition: **(right)** short interval intracortical inhibition (SICI), cortical silent period (CSP) and long interval intracortical inhibition (LICI), and an increase in parameters related to cortical excitation: intracortical facilitation (ICF) and short interval intracortical facilitation (SICF).

The increasing knowledge about cortical hyperexcitability in ALS mainly comes from long and extensive work by the Australian group of researchers lead by Prof. M. Kiernan that introduced the threshold tracking variant of paired pulse (ttppTMS) (Vucic, 2006), along with several new parameters including: short interval intracortical inhibition (SICI), ICF, the long interval intracortical inhibition (LICI) and the short interval intracortical facilitation (SICF), cortical silent period duration (CSP), and index of excitation, all relevant in clinical research, diagnosis and understanding of ALS pathogenesis (reviewed in Rossini et al., 2015; Van den Bos et al., 2018). Reduction or absence of SICI, and increase of ICF have emerged as the two most robust biomarkers of cortical hyperexcitability in ALS (Vucic and Kiernan, 2017), and cortical hyperexcitability was identified as an intrinsic and early feature characterizing both sporadic and familial ALS cases (Vucic et al., 2008), thereby suggesting a similar pathophysiological process (Figure 1). Importantly, reduction of SICI negatively correlates with survival of ALS patients (Shibuya et al., 2016), and increased index of excitation negatively correlated with the Amyotrophic Lateral Sclerosis Functional Rating Scale-Revised score (ALS-FRS) (Van den Bos et al., 2018) highlighting the pathogenic importance of cortical hyperexcitability in ALS. In some familial cases that arise from the mutation of a known gene, cortical hyperexcitability has also been detected pre-symptomatically. This is the case of the *SOD1* mutation carriers (Vucic, 2006; Menon et al., 2015), but not the *FUS* or *C9ORF72* mutation carriers (Blair et al., 2010; Geevasinga et al., 2016).

Interestingly, cortical hyperexcitability (i.e., a combination of decreased SICI and/or CSP and/or increased ICF) was also reported in atypical forms of ALS, such as the primary lateral sclerosis (Geevasinga et al., 2016) characterized by a predominant upper motor neuron (UMN) phenotype, but also, and this is more surprising, the flail leg syndrome (Menon et al., 2016), characterized by a predominant lower motor neuron phenotype with absent or subtle UMN signs. Finally, cortical hyperexcitability has also been demonstrated in patients suffering from FTD (Bae et al., 2016). These studies indicate that cortical hyperexcitability is altogether a typical feature of ALS and closely related diseases, an early biomarker of the disease and a putative prognostic marker, highlighting a potentially important role of cortical circuits dysfunction in ALS pathogenesis.

Short interval intracortical inhibition and cortical silent period are believed to respectively reflect GABA actions on GABA_*A*_ (Di Lazzaro et al., 2012, 2013) and GABA_*B*_ (Chen et al., 2008) receptors, while ICF is believed to reflect activity of the glutamatergic system (Chen et al., 1998). Loss of GABAergic cortical inhibition was further confirmed by Positron Emission Tomography studies with the GABA_*A*_-selective radiotracer [^11^C] Flumazenil that showed binding reductions in sporadic or familial ALS patients compared to controls (Lloyd et al., 2000; Turner et al., 2005; Wicks et al., 2009; Yabe et al., 2012). As of today, it is admitted that dysfunction or degeneration of cortical inhibitory and excitatory components contribute together to cortical hyperexcitability in ALS, and not solely decreased inhibition or increased excitation (Nihei et al., 1993; Ziemann et al., 1996; Enterzari-Taher et al., 1997; Zanette et al., 2002; Karandreas et al., 2009).

### Neuroimaging Studies in ALS

Several functional magnetic resonance imaging (fRMI) studies reported increased functional connectivity (or hyperconnectivity) in the brain of ALS patients compared to controls (Lulé et al., 2007; Douaud et al., 2011; Menke et al., 2016; Schulthess et al., 2016; Agosta et al., 2018), which was also confirmed by electroencephalography (EEG) (Iyer et al., 2015) and resting state magnetoencephalography (MEG) (Proudfoot et al., 2018).

In a longitudinal fMRI study on cortical representation of motor imagery and function during execution task, Lulé et al. (2007) reported a stronger response within premotor and primary motor areas for imagery and execution in ALS patients compared to controls at two time points, and a spread of this increased activity to the precentral gyrus and frontoparietal network at the second time point, indicating a progression over time. Cross-sectional and longitudinal analysis of resting state fMRI (RS-fMRI) data revealed increased functional connectivity successively in the motor, brainstem, ventral attention, and default mode/hippocampal networks (Schulthess et al., 2016), confirming widespread effect on connected brain networks. A recent modeling study based on MRI scans allowed predicting late stage disease burden based on early stage white matter alterations (Meier et al., 2020), further supporting disease spread from the motor cortex along white matter tracts in a spatiotemporal manner (Meier et al., 2020). Together, these studies support disease progression along the corticofugal tracts.

Two scenarios have been proposed to explain increased functional connectivity: first, recruitment of additional brain structures to compensate for the impairment between major nodes of functional networks, and second, loss of inhibitory influence resulting in increased correlation of spontaneous blood-oxygen-level-dependent signals (Douaud et al., 2011).

Increased functional connectivity upon loss of cortical inhibition in ALS is supported by PET studies (Lloyd et al., 2000) and post-mortem histological analyses (Nihei and Kowall, 1993; Nihei et al., 1993; Petri et al., 2003, 2006; Maekawa et al., 2004) (detailed in the section “Cortical Inhibitory GABAergic Interneurons in ALS”), and is in accordance with cortical hyperexcitability revealed by TMS (Vucic, 2006), and further supported by a recent MEG study (Proudfoot et al., 2016).

Few studies on asymptomatic mutation carriers exist that highlight the earliness of cortical impairment in ALS. A functional MEG study reported excess beta-band desynchronization during movement execution in asymptomatic *C9ORF72* mutation carriers (Proudfoot et al., 2016). Similarly, DTI and RS-fMRI analyses revealed increased functional connectivity in asymptomatic *SOD1* or *C9ORF72* mutation carriers (Menke et al., 2016).

While the numerous neuroimaging studies conducted on ALS patients (further reviewed in Chiò et al., 2014; Proudfoot et al., 2019) do not formally demonstrate a cortical origin of ALS, they nevertheless confirm (i) pre-symptomatic cortical dysfunction, (ii) cortical hyperexcitability, and (iii) disease propagation along the cortical connectomes.

## Corticopetal Modulations of Cortical Excitability and ALS

**FIGURE 2:**
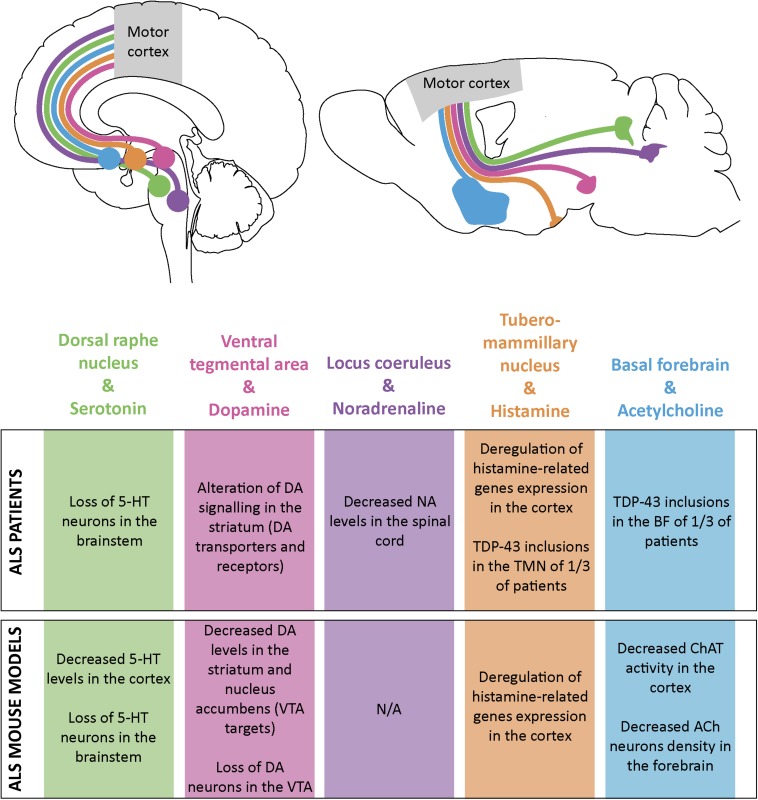
Schematic representation of the monoaminergic and cholinergic systems and their impairments in ALS patients and mouse models. Some monoamine and acetylcholine nuclei send projections to the motor cortex both in human **(top left)** and mouse brain **(top right)**. Alterations in these systems have been found both in ALS patients **(table, upper part)** and ALS mouse models **(table, lower part)**. Dorsal raphe nucleus and its serotonine (5-HT) projections are represented in green; ventral tegmental area (VTA) and dopamine (DA) projections in pink; locus coeruleus (LC) and noradrenaline (NA) projections in purple; tuberomammillary nucleus (TMN) and histamine projections in orange and basal forebrain (BF) and acetylcholine (ACh) projections in blue.

The cerebral cortex sends numerous corticofugal projections to the forebrain, midbrain and hindbrain, and as far as the spinal cord. Similarly, it receives a vast array of corticopetal projections from distant structures that influence its overall activity by the mean of various neuromodulators, the monoamines and acetylcholine (for excellent reviews see Gu, 2002; Vitrac and Benoit-Marand, 2017). This complex regulatory system includes the cholinergic neurons of the basal forebrain, the histaminergic neurons of the tuberomammillary nucleus in the hypothalamus, the serotonergic neurons of the dorsal raphe nucleus, the dopaminergic neurons of the ventral tegmental area (VTA), and the noradrenergic neurons of the locus coeruleus (Figure 2). ALS patients present different levels of alteration of these pathways that could all account for cortical dysfunction in the disease (Figure 2).

### Serotonin and the Dorsal Raphe Nucleus

Serotonin is involved in numerous functions including sleep, attention or sensory information processing. Serotonergic neurons are confined to seven raphe nuclei of the brainstem. Their projections innervate a wide range of structures including the cerebral cortex, hypothalamus, amygdala, hippocampus, cerebellum, and spinal cord. The cerebral cortex is more specifically innervated by the dorsal raphe nucleus (Nestler et al., 2015). In Human, 13 serotonin receptors have been identified amongst which only one is ionotropic and depolarizing. The 12 others are metabotropic with either excitatory or inhibitory effects. In the rodent motor cortex, serotonergic projections are found in every cortical layers (Vertes et al., 1999) and mainly act on excitatory neurons (Santana, 2004). In Human, both acute and chronic administration of paroxetine, a selective serotonergic reuptake inhibitor increased excitability of the motor cortex, as assessed by TMS (Loubinoux et al., 2005). This motor output facilitation was further confirmed by pharmacological and electrophysiological manipulations in the rat (Scullion et al., 2013). It is hypothesized that this effect is mediated by activation of the 5-HT_1A_ receptor expressed by GABAergic interneurons, its hyperpolarizing effect on inhibitory neurons resulting in the release of inhibition on excitatory neurons (Puig et al., 2010; Vitrac and Benoit-Marand, 2017).

Serotonergic neurons also innervate the spinal cord and lower MN in particular. Serotonin action on motoneurons increases persistent calcium current, contributing to the maintenance of their excitability (Heckman et al., 2009). Dentel et al. (2013) showed the degeneration of serotonergic projections to the spinal cord and hippocampus, sometimes accompanied with the loss of serotonergic neuron cell bodies in the brainstem of ALS patients. The *Sod1*^*G86R*^ mouse model of ALS displayed similar serotonergic degeneration, along with a presymptomatic decrease of serotonin levels in the brainstem and spinal cord (Dentel et al., 2013). Loss of serotonin innervation of motoneurons was accompanied by up-regulation of the constitutively active 5-HT_2B/C_ receptors, involved in the development of spasticity (Dentel et al., 2013). Degeneration of serotonergic neurons in *Sod1*^*G86R*^ mice was involved in the spasticity recorded in end-stage animals (Oussini et al., 2017), and treatment of the *Sod1*^*G86R*^ animals with a serotonin inverse agonist abolished end-stage spasticity (Dentel et al., 2013). Importantly, maintenance of serotonergic neurons and projection to the spinal cord in *Sod1*^*G86R*^ animals accelerated appearance of disease onset and worsened motoneuron degeneration without affecting the survival of the animals (Oussini et al., 2017). On the other hand, inhibition of serotonin reuptake by administration of Fluoxetine to 30 day-old presymptomatic or 70 day-old symptomatic *SOD1*^*G93A*^ mice did not affect their survival or motor performances, but transient postnatal Fluoxetine administration proved to be detrimental (Koschnitzky et al., 2014). In all, these studies demonstrated that early stimulation of the serotonergic system in ALS mouse models, either by preventing loss of serotonergic neurons (Oussini et al., 2017), or by treatment of young pups with serotonin reuptake inhibitor may have detrimental effects on disease onset and progression (Koschnitzky et al., 2014). It would be particularly interesting to investigate whether decreased levels of cortical serotonin observed in presymptomatic *Sod1*^*G86R*^ mice (Dentel et al., 2013) has an impact of cortical network function. If similar mechanisms occur in the cortex and spinal cord, it is possible that decreased levels of cortical serotonin could be compensated by the up-regulation of constitutively active serotonergic receptors. This could very well contribute to overall cortical hyperexcitability. Interestingly, decreased binding of the 5-HT_1A_ receptor PET radiotracer [^11^C]-WAY100635 was reported in several cortical areas of sporadic ALS patients compared to controls (Turner et al., 2005; Figure 2), but to our knowledge, binding to the constitutively active 5-HT_2B/C_ receptors in the cerebral cortex of ALS patients has not been reported yet.

### Dopamine and the Ventral Tegmental Area

Dopamine neurons are located in the VTA and the subtantia nigra pars compacta (SNc), both found in the brainstem. Dopaminergic projections reach different cortical areas, including the motor cortex, with a predominance in the frontal cortex, along with other structures: nucleus accumbens, striatum and hippocampus (Nestler et al., 2015). These dopaminergic projections to the motor cortex arise from the VTA and mostly target the deep layers (Vitrac and Benoit-Marand, 2017). While it is not yet clear whom of the excitatory projection neurons and inhibitory interneurons express D1 like and D2 like receptors, stimulation of the VTA of anesthetized rats induced a fast excitatory-inhibitory response of M1 neurons leading to overall facilitation of motor output to forelimb muscles (Kunori et al., 2014). However, depressions of cortical neuronal activity in response to dopamine were also reported (Gu, 2002; Vitrac and Benoit-Marand, 2017). A TMS study carried on healthy individuals showed that the receptor agonist cabergoline, thought to act mostly on D2 and D3, decreased cortical excitability (by increasing SICI) (Korchounov et al., 2006). Similarly, enhanced cortical inhibition by dopamine receptor agonists pergolide and bromocriptine, and decreased cortical inhibition by dopamine receptor antagonist haloperidol were previously reported (Ziemann et al., 1996). These data suggest that dopamine may act as a break to motor cortex excitability in humans.

This hypothesis is further supported by data obtained from Parkinson’s disease patients. Parkinson’s disease arises from the degeneration of dopaminergic neurons of the substantia nigra pars compact (Michel et al., 2016), but loss of neurons in the VTA has also been reported (Alberico et al., 2015). Interestingly, Parkinson’s disease was also associated with cortical hyperexcitability mostly arising from decreased inhibition which can be partially reversed by dopaminergic therapies (Ridding et al., 1995; Vucic and Kiernan, 2017). Dopaminergic impairment (Martorana, 2014) and cortical hyperexcitability (Vucic and Kiernan, 2017) have also been documented in Alzheimer’s disease. Similarly to the effect of dopaminergic therapies on cortical excitability in Parkinson’s disease, L-Dopa restored normal intra-cortical inhibition in Alzheimer patients (Martorana, 2014). These data suggest that dopamine is involved in the modulation of cortical activity and that dopaminergic system impairment can lead to loss of cortical inhibition, and potentially to cortical hyperexcitability.

Less is known about potential dopaminergic impairment in ALS. Single photon emission computed tomography (SPECT) studies revealed a decrease of dopaminergic terminals in the putamen and caudate nucleus of ALS patients (Borasio et al., 1998), and a decrease of striatal D2 receptors (Vogels et al., 2000) without neuronal loss. Interestingly Vogels et al. (2000) attributed the loss of D2 receptor to increased glutamatergic signaling from the cerebral cortex, via the corticostriatal pathway. Fu et al. (2017) also reported on the reduced binding of ^18^F-fallypride (D2/D3 antagonist) in different regions of the cerebral cortex. However, these studies did not investigate or report on dopaminergic signaling onto the motor cortex itself. In end-stage *SOD1*^*G93A*^ mice, Kostic et al., 1997 reported decreased levels of dopamine in the caudate-putamen and nucleus accumbens, together with a loss of dopaminergic neurons in both the VTA and the subtantia nigra pars compacta. In the same mouse line, MRI analysis recently confirmed VTA impairment and immunohistology revealed a loss of up to 50% of dopaminergic neurons in the VTA, and vacuolization of the remaining ones at disease end-stage (Jouroukhin et al., 2013; Figure 2). Given the likely inhibitory role of dopamine on cortical networks, and the clinical and preclinical arguments indicating an impairment of the dopaminergic system in ALS patients and mouse models, it would be interesting to determine whether such impairment could also affect the motor cortex and contribute to its hyperexcitability.

### Noradrenaline and the Locus Coeruleus

The large majority of noradrenaline (or norepinephrine) is produced by neurons located in the locus coeruleus, a nucleus lying on the floor of the 4th ventricle in the rostral pons. A small proportion of the noradrenaline also arises from the brainstem and the lateral tegmental regions (Nestler et al., 2015; Schwarz and Luo, 2015). The only source of cortical noradrenaline is the locus coeruleus, which also innervates other regions of the forebrain, the brainstem, the cerebellum and the spinal cord. In the motor cortex, noradrenergic projections innervate every cortical layers but a greater density is found in layer I and layer VI (Agster et al., 2013). Noradrenaline acts on adrenergic receptors, which are G-protein coupled receptors. This family is composed of nine members that belong to the α- or β-subfamilies, both types found in the cerebral cortex. Whereas all β-receptors are excitatory, α-receptors can be excitatory or inhibitory, depending on the G-protein. Treatment with selective noradrenaline reuptake inhibitor or α_2_ antagonist demonstrated in TMS that noradrenaline enhanced cortical excitability in healthy humans (Plewnia et al., 2001).

Pathological studies revealed neurofibrillary tangles in the locus coeruleus of ALS patients (Orrell et al., 1995) but apparently no TDP-43 pathology (Brettschneider et al., 2013) or cell loss (Hoogendijk et al., 1995). However, liquid chromatography analysis of spinal cord samples from ALS patients revealed reduced levels of noradrenaline compared to controls (Bertel et al., 1991), suggesting that noradrenergic impairment could occur in ALS, without necessary loss of neurons in the locus coeruleus (Figure 2). If little is known about the state of the locus coeruleus and the noradrenergic system in ALS, this is not the case under other neurodegenerative conditions, and more particularly in PD and AD (for excellent review, see Marien et al., 2004) where neuronal loss in the locus coeruleus is more important than in the substantia nigra in PD, or the nucleus basalis in AD (Zarow et al., 2003).

### Histamine and the Tuberomammillary Nucleus

In the brain, histamine is synthesized exclusively by neurons located in the tuberomammillary nucleus which lies within the posterior hypothalamus (Nestler et al., 2015), and is implicated in sleep–wake cycle, nociception, motor circuits, satiety signaling, and neuroimmune functions (Panula and Nuutinen, 2013). Histaminergic neurons project both in the brain and the spinal cord with dense projections to the cerebral cortex. Histamine acts as a neurotransmitter and activates four subfamilies of metabotropic receptors, H_1__–__4_ leading either to neuronal depolarization or hyperpolarization of neurons, depending on the identity of the G-protein (Haas and Panula, 2003; Shan et al., 2015). In the rodent motor cortex, histaminergic projections are found in every cortical layers (Panula et al., 1989) and can act both on excitatory and on inhibitory neurons. In addition to direct connections to the cerebral cortex, histamine may also indirectly influence cortical function via activation of cholinergic basal forebrain neurons and induction of acetylcholine release in the cortex (Cecchi et al., 2001; Zant et al., 2012). Finally, treatment with H_3_ receptor inverse agonist was shown to trigger increased levels of serotonin, noradrenaline and dopamine in the rat prefrontal cortex highlighting a potential central role of histamine in regulating the function of several cortical areas (Flik et al., 2015).

Detailed analysis of the hypothalamus of ALS patients revealed the presence of TDP-43 pathology in 5/28 cases (Cykowski et al., 2014), suggesting that neurons of the tuberomammillary nucleus may not be a primary target of the TDP-43 pathology, and may maintain, in most cases their function. The group of Sebastiano Cavallaro characterized the transcription profile of the motor cortex from sporadic ALS patients compared to healthy individuals (Aronica et al., 2015). Further data mining allowed them to identify the significant up-regulation of the genes involved in histamine synthesis (histidine decarboxylase, HDC) and catabolism (histamine N-methyl transferase and diamine oxydase, HNMT) (Apolloni et al., 2017). These data were further corroborated by western blot analyses of *SOD1*^*G93A*^ mouse cerebral cortex showing transient symptomatic up-regulation of HDC and HNMT, along with early up-regulation of H_4_ and late up-regulation of H_1_ receptors (Apolloni et al., 2017). Together these studies suggest that in ALS patients and mouse models the histaminergic system may be overall up-regulated, similarly to what has been reported for Parkinson’s or Huntington’s diseases (Shan et al., 2015). Importantly, symptomatic treatment of *SOD1*^*G93A*^ mice with histidine, the brain-permeable precursor of histamine, improved motor performance and increased survival (Apolloni et al., 2019). Whether this beneficial effect can solely be attributed to an anti-inflammatory response of cortical and spinal microglia, or to additional improvement of cortical and spinal network functions remains to be determined, but this seminal study strongly highlights the histaminergic system as a new therapeutic target in ALS (Apolloni et al., 2019; Figure 2). In addition to its direct effect on cortical cells and networks, histamine’s indirect effects via serotonin, noradrenaline, and dopamine (Flik et al., 2015), all known to also modulate motor cortex activity (Vitrac and Benoit-Marand, 2017), make this monoamine a particularly interesting neuromodulator to follow in the context of ALS cortical dysfunctions and therapeutic intervention.

### Acetylcholine and the Basal Forebrain

In the human brain, the vast majority of cholinergic neurons are found clustered in eight small nuclei located in the basal forebrain and the brainstem, but scattered cholinergic neurons are also present in different brain regions (Nestler et al., 2015). Cholinergic projections to the cerebral cortex arise mostly from the nuclei of the basal forebrain, and more particularly the nucleus basalis and the substantia innominata, and follow a precise and complex topographical organization (reviewed in Ballinger et al., 2016). In the cerebral cortex, acetylcholine acts both on excitatory pyramidal neurons and inhibitory interneurons. In addition, acetylcholine acts on two types of receptors: the ionotropic nicotinic receptors and the metabotropic muscarinic receptors. While binding of acetylcholine on nicotinic receptors induces depolarization, facilitating the excitation of the post-synaptic cell, binding of acetylcholine on muscarinic receptors induces either depolarization or hyperpolarization depending on the G-protein coupled to the receptor, and leads either to excitation or inhibition of the post-synaptic neuron (Ballinger et al., 2016). The duality of effects – excitatory or inhibitory –, along with the duality of targeted neuronal populations – excitatory or inhibitory –, and the variety of responses across cortical layers reflects the subtlety of acetylcholine action on cortical networks (Ballinger et al., 2016). Overall, if many studies converge toward a slow modulatory effect of acetylcholine resulting in an increased excitability of the targeted networks, recent work indicates that transient and faster kinetics of cholinergic signaling also exist (Sarter et al., 2014).

Basal forebrain cholinergic neurons have been mostly studied for their physiological role in sensory detection, attention, learning and memory, and their pathological contribution to cognitive decline (for review see Ballinger et al., 2016), but much less attention has been given to their effect on the motor cortex, or in the context of ALS. Kuo et al. (2007) demonstrated in the human motor cortex that acetylcholine could either increase or decrease cortical excitability, according to the basal stimulation conditions of the network, improving signal-to-noise ratios and refining information processing. Cykowski et al. (2014) reported the presence of cytoplasmic inclusions of TDP-43 in the basal forebrain and hypothalamus of one-third of ALS patients, suggesting that impairment of cholinergic neurons may occur in ALS. However, radioactive labeling of muscarinic receptors in the cerebral cortex did not show any difference between ALS patients and controls (Gredal et al., 1996), suggesting that basal forebrain alteration might not impact muscarinic receptors density in the cortex. Finally, symptomatic *SOD1*^*G93A*^ mice displayed decreased numbers of cholinergic neurons in the basalis nucleus of the basal forebrain, that could account at least partly for the overall decrease in acetylcholine transferase activity measured in the cerebral cortex of these same animals (Crochemore et al., 2005; Figure 2). Thus, while little is known about a possible contribution of acetylcholine and basal forebrain cholinergic neurons to physiological motor cortical network function, and dysregulation in ALS, the subtle role of this neurotransmitter in other cortical areas and its implication in other neurodegenerative conditions such as Alzheimer’s or Parkinson’s diseases provide appealing background to push forward the investigation of the cholinergic system in the field of ALS.

All above-mentioned neuromodulators can impact the activity of the motor cortex (Vitrac and Benoit-Marand, 2017). Some of them have already been associated with neurodegenerative conditions, and thus represent possible contributing factors to cortical hyperexcitability in ALS (Figure 2).

## Morphological and Functional Alterations of Cortical Excitatory and Inhibitory Neurons in ALS

Whether of extra- or intra-cortical origin, or both, cortical excitation/inhibition (E/I) imbalance results from a dysfunction of the cortical circuits, i.e., from one or several of the cellular components of the cerebral cortex: the excitatory glutamatergic projection neurons, the inhibitory GABAergic interneurons, and possibly the astrocytes, for their contribution to the tripartite synapse and role in neuronal network development and maintenance (Farhy-Tselnicker and Allen, 2018). Analysis of the contribution of these cellular populations and their numerous sub-populations is limited in patients, because the techniques that can be applied on alive persons remain at the scale of the network/structure, and because analysis of post-mortem tissues, while extremely relevant, provides the snapshot of an exhausted network, and little information about the succession of events that drove it there. This is where preclinical models, and particularly rodent models of the disease can be extremely useful, proven that they recapitulate a few essential hallmarks of the disease.

### Evidences for Early Cortical Impairment in Rodent Models of ALS

If the spinal cord, the MN and the skeletal muscle occupy a prominent place in the landscape of preclinical ALS research, a few recent seminal studies contributed to shed light on a possible early role of the cerebral cortex and its neuronal populations in ALS pathophysiology. Indeed, several mouse models of the disease recapitulate CSN or subcerebral projection neuron (that comprise CNS) degeneration. This is true for several mutant *SOD1* mouse models (Zang and Cheema, 2002; Ozdinler et al., 2011; Yasvoina et al., 2013; Marques et al., 2019) but also *C9ORF72* (Liu et al., 2016) *PFN1* (Fil et al., 2016), and *TARDBP* (Wegorzewska et al., 2009; Herdewyn et al., 2014). In several of these models, CSN loss precedes motor symptoms and neuromuscular junction denervation (Zang and Cheema, 2002; Ozdinler et al., 2011; Yasvoina et al., 2013; Marques et al., 2019), indicating that cortical alterations may take place very early in these models. Recently, we ran a comprehensive spatiotemporal analysis of CSN degeneration in the *Sod1*^*G86R*^ mouse model of ALS, and showed that loss of CSN preceded loss of MN but also neuromuscular junction denervation, and weight loss (Marques et al., 2019). Early loss of CSN was accompanied with pre-symptomatic occurrence of hyperreflexia, a component of the UMN syndrome (Burg et al., 2019). Finally, CSN and MN degenerations were also somatotopically related, further suggesting that early cortical impairment may negatively influence MN function and survival (Marques et al., 2019). Together, these studies indicate that loss of CSN or subcerebral projection neurons not only occurs in mouse models of ALS, but also that, when temporarily assessed, precedes MN degeneration, suggesting that alteration of cerebral cortex is a very early event on the time scale of disease progression.

First genetic manipulation in the cerebral cortex of a rat model of ALS further informed on the role of this structure in disease onset and progression. Thomsen et al. (2014) knocked-down a mutant *SOD1* transgene in the posterior motor cortex of the *SOD1*^*G93A*^ rat model of ALS, using an AAV9 virus to selectively transduce neurons. AAV9-SOD1-shRNA injections delayed disease onset and extended survival without affecting disease duration (Thomsen et al., 2014). Recently, we experimentally tested the corticofugal hypothesis of ALS, by generating a mouse line ubiquitously overexpressing the murine *Sod1*^*G86R*^ transgene, a condition sufficient to mimic ALS symptoms and premature death (Ripps et al., 1995), and knocked-out for the gene *Fezf2*, a condition sufficient to prevent the specification of subcerebral projection neurons (Molyneaux et al., 2005). Absence of subcerebral projection neurons, and in particular of CSN, was sufficient to delay disease onset, limit weight loss and motor decline and extend survival of the animals, providing a first experimental support to the corticofugal hypothesis (Burg et al., 2019). Together, these studies provide evidence for an early contribution of cortical dysfunctions in ALS.

### Cortical Excitatory Glutamatergic Neurons, and Corticospinal Neurons in ALS

**FIGURE 3:**
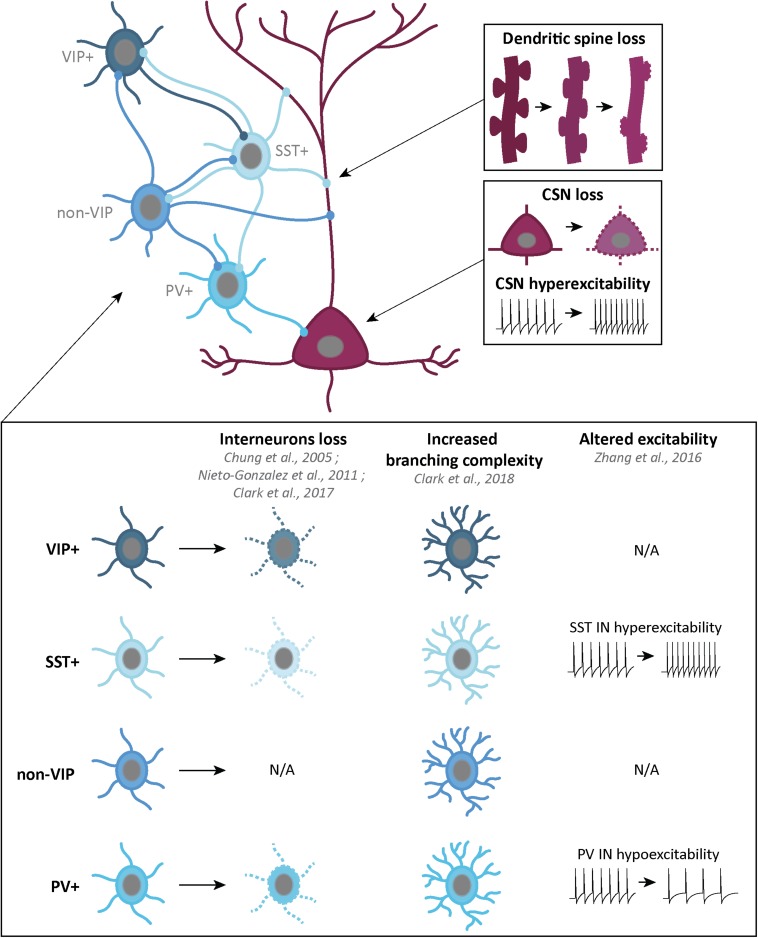
Schematic representation of the corticospinal neurons and GABAergic interneurons, and their alterations in ALS patients or mouse models. Corticospinal neurons (CSN) are excitatory glutamatergic neurons (dark pink) which, in ALS, present progressive dendritic spine loss **(top right)**, cell loss and hyperexcitability of the remaining neurons **(middle right)**. GABAergic interneurons (IN) are inhibitory neurons (four shades of blue) which can be classified in four groups: vasointestinal peptide (VIP) IN, somatostatin (SST) IN, non-VIP IN and Parvalbumin (PV) IN. Some alterations in IN density, branching complexity and excitability have been found in different ALS mouse models and ALS patients (bottom part).

In Humans, loss of CSN, or upper motor neurons (UMN), or Betz cells, and of their projections within the CST, results in appearance of the UMN syndrome, a series of symptoms including decreased motor control, altered muscle tone and strength, hyperreflexia, spasticity, and clonus (Ivanhoe and Reistetter, 2004), and allows the diagnosis of ALS, when combined with signs of bulbar or spinal MN degeneration (muscle denervation). Quantification of the progressive loss of Betz cells can be achieved in alive patients with the triple stimulation technique which estimates the proportion of motor units that can be stimulated by TMS (Wang et al., 2019), but to our knowledge, the technique has not been used yet for longitudinal analysis of CSN loss in ALS, or on pre-symptomatic patients. Together with a “pallor of the CST” (Charcot, 1874) that runs laterally in Human spinal cord, post-mortem analyses of ALS patients revealed a depletion of the giant Betz cells (CSN) of about 50–60%, and a shrinkage of the remaining Betz cell bodies (Nihei et al., 1993; Figure 3).

As already mentioned, various mouse models of the disease present with a loss of CSN or of cortical layer V subcellular projection neurons at disease end stage, such as the *C9-BAC* (Liu et al., 2016), the *hPFN1*^*G118V*^ (Fil et al., 2016), the *TDP-43^*A315V*^* mouse models (Wegorzewska et al., 2009; Herdewyn et al., 2014) and the *TDP-43^*G298S*^* (Müller et al., 2019). *SOD1* mouse, generated earlier and further analyzed display a progressive loss of CSN that starts pre-symptomatically (Zang and Cheema, 2002; Ozdinler et al., 2011; Yasvoina et al., 2013; Marques et al., 2019; Figure 3). These studies reflect early alterations of the motor cortex of mouse models of the disease. In the context of overall cortical hyperexcitability, presymptomatic loss of a subpopulation of cortical excitatory neurons may reflect the consequence of an over-active network, rather than the origin of this E/I imbalance. Thus paralleled longitudinal analyses to compare the rate of CSN loss and the progression of cortical hyperexcitability, either on patients, or in mouse models of the disease, would be particularly informative.

In mice, CSN and other layer V subcerebral projection neurons display, prior to their degeneration, morphological alterations or electrophysiological dysfunctions that reflect either their hyperexcitable state or altered excitatory and inhibitory inputs, or both. In young and presymptomatic *SOD1*^*G93A*^ mice, CSN and other layer V subcerebral projection neurons present selective apical dendrite degeneration, together with a lack of spines and a vacuolation, but no impairment of the basal dendrites (Jara et al., 2015), before they start degenerating. Progressive spine loss was also reported in apical and basal dendrites of layer V subcerebral projection neurons located in the motor cortex of presymptomatic *TDP-43^*A315V*^* mice (Handley et al., 2018; Figure 3). Such features were also reported in the remaining Betz cells of sporadic and familial ALS cases (Hammer et al., 1979; Genç et al., 2017). In the motor cortex of *TDP-43^*Q331K*^* mice instead, Fogarty et al. (2016) reported increased spine densities along the proximal and distal compartments of apical and basal dendrites of layer V subcerebral projection neurons. While the two TDP-43 mouse models apparently display opposite spine phenotypes, it is worth mentioning that both affect the formation or maintenance of dendritic spines, in accordance with an emerging role of TDP-43 and other RNA-binding protein in synaptic integrity (Sephton and Yu, 2015).

Such morphological abnormalities are accompanied by changes in electrophysiological properties. Initial work from the team of Cristina Zona reported intrinsic hyperexcitability of primary cortical neurons from *SOD1*^*G93A*^ embryos, compared to controls, as a result of increased persistent voltage-dependent Na^+^ current (I_*NaP*_) (Pieri et al., 2009). Patch-clamp methods on brain slices further demonstrated hyperexcitability of layer V subcerebral projection neurons from the motor cortex of presymptomatic (21 and 30 day-old) *SOD1*^*G93A*^ mice (Saba et al., 2015). Kim et al. (2017) patched retrogradely labeled CSN and callosal projection neurons (CPN) present in the layer V of *SOD1*^*G93A*^ motor cortex, and demonstrated increased intrinsic excitability of both neuronal types (Kim et al., 2017). Layer V subcerebral projection neurons displayed increased excitatory synaptic inputs in the presymptomatic *TDP-43^*Q331K*^* mice (Fogarty et al., 2016), but unchanged (P21) or decreased (P60) frequency of excitatory synaptic transmission in the presymptomatic *TDP-4343^*A315T*^* mice (Handley et al., 2018; Figure 3). However, P21 layer V subcerebral projection neurons from the *TDP-43^*A315T*^* mice presented decreased inhibitory synaptic transmission resulting in hyperexcitability that was sustained throughout disease progression (Zhang et al., 2016). Interestingly, similar observations had been made previously in the Wobbler mouse model of ALS (Nieto-Gonzalez et al., 2010).

### Cortical Inhibitory GABAergic Interneurons in ALS

Cortical GABAergic interneurons (INs) can be differentiated by molecular markers, firing pattern and cortical layers organization (Markram et al., 2004). Recently, a classification into three broad subgroups has been proposed: parvalbumin (PV)-positive INs (∼40%), somatostatin (SST)-positive INs (∼30%) and 5-HT_3A_R-positive INs (∼30%) (Tremblay et al., 2016). Other markers also overlap with these groups. Neuropeptide Y (NPY), is expressed in a subpopulation of SST+ INs, and 5-HT_3A_R+ INs group can be divided into vasointestinal peptide (VIP)-positive and non-VIP INs. Finally, while some SST+ INs and VIP+ INs share calretinin (CR) as a marker, some SST+ and non-VIP INs share both nitric oxide synthase (NOS) and Reelin markers (Tremblay et al., 2016; Figure 3).

Interneurons inhibit not only projection neurons but also other interneurons. Layers V projection neurons, including CSN, receive GABAergic inputs from non-VIP INs on their apical dendrite in layers I and II/III, and on their basal dendrites in layers V/VI. SST+ INs inputs only come from layers V/VI. Finally, layers V/VI PV+ INs provide somatic inhibition. VIP+ INs are specifically involved in SST+ INs inhibition (Figure 3).

Impairment of GABAergic inhibition, starting with the loss of cortical INs, represents a simple way to explain cortical hyperexcitability in ALS. PET studies conducted on ALS patients with [^11^C]flumazenil revealed a decreased density of GABA_*A*_ receptors (Lloyd et al., 2000). Whether this reflects a loss of cortical (excitatory and/or inhibitory) neurons, or a down-regulation of post-synaptic GABA_*A*_ receptor expression, or both, is suggestive of an overall decreased cortical inhibition. Post-mortem analyses revealed decreased GABA_*A*_ mRNA levels (Petri et al., 2003, 2006) significant loss of calbindin (CB) and PV immunoreactivity in the motor cortex of ALS patients, and atrophy of NPY-positive INs (Nihei and Kowall, 1993; Nihei et al., 1993; Maekawa et al., 2004). Decreased PV immunoreactivity could arise from a genuine neuronal loss, but also from decreased expression. Interestingly, decreased levels of PV modifies the dynamic of burst discharge (Albéri et al., 2013) and absence of PV increases the susceptibility to Pentylenetetrazole (PTZ)-induced seizures in mouse (Schwaller et al., 2004), suggesting that both loss of PV-positive neurons, or decreased levels of the protein may affect proper functioning of the cortical network (Figure 3).

Quantifications of the various IN populations in the multiple mouse models of ALS gave different results. In the primary motor cortex of pre-symptomatic Wobbler mice, Nieto-Gonzalez et al. (2010) observed decreased densities of PV-positive and SST-positive INs, together with a reduced density of GABAergic synaptic boutons. In the *SOD1*^*G93A*^ mouse model, classical quantification methods reported levels of PV+ INs similar to wild-type animals (Ozdinler et al., 2011; Clark et al., 2017). However, the Voronoi tessellation method revealed increased numbers of PV-positive INs, selectively in the motor and somatosensory areas, from pre-symptomatic ages to disease end-stage (Minciacchi et al., 2009). Early morphological alteration and late loss of upper layer CR-positive INs, together with a late increase of NPY immunoreactivity were also reported in *SOD1*^*G93A*^ mice (Clark et al., 2017). Interestingly, the authors suggested that increased NPY expression in various IN populations could reflect the need to express neuroprotectants in a context of altered and toxic cortical activity (Clark et al., 2017). In *TDP-43^*A315T*^* mice, Zhang et al. (2016) reported pre-symptomatic decrease followed by symptomatic loss of SST-positive neurons (Figure 3). Together, the data indicate that GABAergic INs undergo modifications in various mouse models of ALS, suggesting impairments of the inhibitory circuits. Above cell numbers, levels of calcium-binding proteins, such as PV, or of neuroprotective neuropeptides, such as NPY, need to be carefully assessed to further evaluate the inhibitory component of the cortical network. Interestingly, SST-positive neurons from different cortical areas were reported to receive dopaminergic, noradrenergic, and serotonergic innervations, and SST to play the role of co-transmitter for GABA, thus involved in modulating surrounding neurons (for reviews see Liguz-Lecznar et al., 2016; Riedemann, 2019).

Kim et al. (2017) reported that PV-positive INs from neonatal and symptomatic *SOD1*^*G93A*^ mice were hyperexcitable, similar to excitatory layer V CSN and CPN. Contrastingly, Zhang demonstrated that PV-positive INs were hypoactive, while SST-positive INs were hyperactive in the *TDP-43^*A315T*^* mouse model of ALS (Zhang et al., 2016). The authors elegantly demonstrated that hyperactivity of SST-positive INs was responsible for hypoactivity of PV-positive INs, and, in turn, for hyperexcitability of layer V subcerebral projection neurons, which could be restored to normal excitability by genetic ablation of SST-positive INs (Zhang et al., 2016; Figure 3). A better understanding and characterization of cortical INs families, inputs and outputs, as well as morphology and electrophysiology, will be essential in the future to better dissect the mechanisms underlying cortical hyperexcitability in ALS.

## Altered Gene Expression Related to Cortical Circuits Dysfunction in ALS

Neuronal excitability relies first on the establishment of membrane potential, i.e., differential concentrations of cations on each side of the plasma membrane, due to the presence of ATP-dependent sodium potassium pumps (Na^+^K^+^ pumps). Neuronal excitability is the ability of a neuron to respond to stimuli by rapid change in membrane potential, a phenomenon that requires the selective opening of specific ion channels. Thus, amounts and compositions of ion channels are directly related to neuronal excitability, and neuronal hyperexcitability can also be seen as the result of altered expression of these protein families, along with neurotransmitter receptors and other synaptic proteins.

In order to better understand the pathologic cascade leading to the neuronal degeneration in the cerebral cortex, a few studies interrogated the transcriptomic alterations in post-mortem cortex (motor or frontal) from ALS patients (Wang et al., 2009; Lederer et al., 2007; Aronica et al., 2015; Prudencio et al., 2015; Andrés-Benito et al., 2017), or individual populations of cells purified from the mouse cerebral cortex (Kim et al., 2017; Marques et al., 2019). Given the emerging role of altered RNA metabolism – and thus altered protein expression – as a common pathological mechanism of ALS, these seminal studies may point to transcriptomic alterations that could possibly underlie cortical hyperexcitability in ALS.

### Ion Homeostasis and Transportation

First microarray analysis of the motor cortex of sporadic ALS patients compared to control individuals revealed a small number of deregulated genes (Lederer et al., 2007). Amongst others, the study reported on the down-regulation of *ATP1A3* (*ATPase Na^+^/K^+^ transporting subunit alpha 3*), that belongs to the family of P-type cation transport ATPases, and contributes to establishing and maintaining the electrochemical gradients of Na^+^ and K^+^ ions across the plasma membrane. Because mutations of *ATP1A3* cause rapid-onset dystonia parkinsonism (RDP), alternating hemiplegia of childhood (AHC), or early infantile epileptic encephalopathy (EIEE), the later two being characterized by seizures (Arystarkhova et al., 2019), its down-regulation in ALS patients could have repercussions on cortical excitability. In the same study, Lederer et al. (2007) also reported on the down-regulation of *KCNC2* (*potassium voltage-gated channel subfamily C member 2*), while similar microarray analyses conducted on motor and sensory cortex (Wang et al., 2009), or on motor cortex of ALS patients (Aronica et al., 2015) revealed the up-regulation of *KCNIP2* (potassium voltage-gated channel interacting protein 2) and of *SCN7A* (sodium voltage-gated channel alpha subunit 7), respectively, further supporting altered neuronal excitability and action potential firing. Down-regulation of *SLC12A5* (*solute carrier family 12 member 5*), a K^+^/Cl^–^ co-transporter, is also noticeable (Lederer et al., 2007). During development, increased expression of *SLC12A5*, formerly known as *KCC2*, lowers intracellular chloride concentrations below the electrochemical equilibrium potential, allowing GABA’s action on postsynaptic components to switch from excitatory to inhibitory (Schulte et al., 2018). Thus, decreased expression of *SLC12A5* in the pathological context of ALS could partly reverse this effect and GABA-mediated inhibition of cortical networks.

The protein hormone *Adiponectin* (*ADIPOQ*) and its receptors R1 and R2 (*ADIPOR1* and *ADIPOR2*) were found down-regulated in the motor cortex of ALS patients compared to controls (Aronica et al., 2015). In the paraventricular nucleus of the hypothalamus, adiponectin and its receptors were shown to regulate neuronal excitability via their modulation of different potassium currents (Hoyda and Ferguson, 2010). In the hippocampus, *ADIPOR2* deletion leads to hyperexcitability of the dentate gyrus neurons (Zhang et al., 2016). Finally, adiponectin has been related to several disorders of the central nervous system, such as stroke, Alzheimer’s disease, Parkinson’s disease and Multiple sclerosis (for review see Baranowska-Bik and Waszkiewicz-Hanke, 2017; Bloemer et al., 2018).

### Glutamatergic and GABAergic Components

Wang et al. (2009) reported up-regulations of the glutamate receptor *GRIK1* (*glutamate ionotropic receptor kainate type subunit 1*), and of the postsynaptic density scaffolding protein *HOMER 3* (*homer scaffold protein*), which binds group I metabotropic glutamate receptors, amongst numerous other proteins. Aronica et al. (2015) instead reported on the up-regulation of *GRIA1* in a subgroup of ALS patients, and down-regulation of several ionotropic and metabotropic glutamate receptors, *GRIN1*, *GRIN2A*, *GRIN2D*, *GRIA2*, and *GRIA3*, in a second subgroup of patients. This was accompanied with the down-regulation of six subunits of the GABA_*A*_ receptor in one subgroup of patients (Aronica et al., 2015). These transcriptomic changes in the post-mortem motor cortex of ALS patients could reflect the broad neuronal loss that occurred prior to death. In this regard, the study by Andrés-Benito et al. (2017) is particularly interesting because it was conducted on the frontal cortex of ALS patients that showed no sign of FTD, and for which no neuronal loss in the frontal cortex was suspected. Thus, the analysis could potentially provide a molecular snapshot of cortical hyperexcitability prior to neurodegeneration. In this study, numerous glutamate receptors and transporters were found up-regulated (Andrés-Benito et al., 2017). This was the case of *GRIA1*, which codes for the ionotropic glutamate receptor AMPA 1, *GRIN2A* and *GRIN2B*, which code for NMDA receptors, and *GRM5*, which codes for the glutamate metabotropic receptor 5, along with the glutamate transporters *SLC1A2* and *SLC17A7* (Andrés-Benito et al., 2017). Interestingly, *GAD1*, that encodes the glutamate carboxylase 1, that synthesizes GABA from glutamate, and the GABA_*A*_ and GABA_*B*_ receptors subunits *GABRD*, *GABRB2*, and *GABBR2* were also increased (Andrés-Benito et al., 2017). Increased expression of components of the glutamatergic system are in agreement with overall increased cortical excitability, and increased expression of components of the GABAergic system could be interpreted as an attempt to counteract increased excitation.

### Altered Gene Expression in Mouse Models of ALS Suggest Possible Cortical Hyperexcitability in Rodents

While to our knowledge TMS has not been employed in mouse models of ALS and broad cortical hyperexcitability has not been demonstrated in these animals, Kim et al. (2017) reported intrinsic hyperexcitability of cortical neurons, and particularly layer V subcerebral projection neurons, but also CPN and populations on interneurons. Using RNAseq on purified sub-populations of cortical neurons, they demonstrated that intrinsic hyperexcitability of corticospinal neurons (CSN) and CPN from postnatal *SOD1*^*G93R*^ mice was accompanied by changes of expression of several voltage-gated Na^+^ and K^+^ channels, GABA and glutamate receptors (Kim et al., 2017). Interestingly, the sets of differentially regulated genes were different between the two neuronal populations, and the variations of common genes were sometimes opposite (Kim et al., 2017). This is in agreement with the different molecular identities of the two investigated populations, but may also reflect different strategies to deal with hyperexcitability, depending on the position within the cortical networks.

## Concluding Remarks

A growing number of evidences point to the cerebral cortex as the origin of ALS and suggest a corticofugal propagation of the disease. In this context, the earliness of cortical hyperexcitability in ALS patients, and the recent demonstration that it is sufficient to trigger TDP-43 pathology, suggest that cortical E/I unbalance may represent *per se* a particularly relevant therapeutic target. Because numerous extrinsic (i.e., non-cortical) and intrinsic (i.e., cortical) components contribute to the fine-tuning of cortical excitability and cortical network proper functioning, the possible candidates to cortical hyperexcitability are numerous, and combined impairments are very likely. To further unravel the mechanisms behind cortical hyperexcitability in ALS, mouse models that recapitulate this typical hallmark of the disease are needed, or, more simply, current mouse models of the disease should be tested for possible cortical hyperexcitability. These will in turn allow assessing various therapeutic strategies to restore proper cortical excitability, and to determine the impact of such intervention on direct corticofugal targets, and more broadly on disease onset and progression. These first steps may in turn pave the way to a new era of treatment in the field of ALS, and potentially other neurodegenerative diseases.

## Author Contributions

AB, GS-L, JS-Z, TB, and CR analyzed the data of the literature and wrote the manuscript. AB and CR designed the figures and AB elaborated them. All authors approved the publication of the manuscript.

## Conflict of Interest

The authors declare that the research was conducted in the absence of any commercial or financial relationships that could be construed as a potential conflict of interest.
